# Author Correction: Experimental and theoretical model for the origin of coiling of cellular protrusions around fibers

**DOI:** 10.1038/s41467-023-44423-4

**Published:** 2023-12-21

**Authors:** Raj Kumar Sadhu, Christian Hernandez-Padilla, Yael Eshed Eisenbach, Samo Penič, Lixia Zhang, Harshad D. Vishwasrao, Bahareh Behkam, Konstantinos Konstantopoulos, Hari Shroff, Aleš Iglič, Elior Peles, Amrinder S. Nain, Nir S. Gov

**Affiliations:** 1 Department of Chemical and Biological Physics, Weizmann Institute of Science, Rehovot, 7610001 Israel; 2 Department of Mechanical Engineering, Virginia Tech, Blacksburg, VA 24061 USA; 3 Department of Molecular Cell Biology, Weizmann Institute of Science, Rehovot, 7610001 Israel; 4 Laboratory of Physics, Faculty of Electrical Engineering, University of Ljubljana, Ljubljana, Slovenia; 5 Advanced Imaging and Microscopy Resource, National Institutes of Health, Bethesda, MD USA; 6 Department of Chemical and Biomolecular Engineering, Johns Hopkins University, Baltimore, MD USA; 7 Laboratory of High Resolution Optical Imaging, National Institute of Biomedical Imaging and Bioengineering, National Institutes of Health, Bethesda, MD USA; 8 Present Address: Institut Curie, PSL Research University, CNRS, UMR 168 Paris, France

**Keywords:** Membrane biophysics, Cell adhesion, Membrane structure and assembly

Correction to: *Nature Communications* 10.1038/s41467-023-41273-y, published online 12 September 2023

The original version of this Article contained an error in the Acknowledgments, which incorrectly omitted the following: “E.P. acknowledges the Israel Science Foundation, Dr. Miriam and Sheldon G. Adelson Medical Research Foundation, and Estate of Paula Vial Lempert, Sassoon and Marjorie Peress, the Estate of Gerda Wassermann, and the Estate of Dr. Sylvia Brody Axelrad”. This has been corrected in both the PDF and HTML versions of the Article.

