# Supplementary material for: Experimental and theoretical model for the origin of coiling of cellular protrusions around fibers
Source: Nat Commun. 2023 Sep 12;14:5612. doi: 10.1038/s41467-023-41273-y (PMC10497540; doi:10.1038/s41467-023-41273-y)
Supplement: Supplementary file 1 — Supplementary Information [file 41467_2023_41273_MOESM1_ESM.pdf]

Supplementary Information for
Experimental and Theoretical model for the origin of coiling of cellular protrusions around fibers

Raj Kumar Sadhu[1ab], Christian Hernandez-Padilla[2], Yael Eshed Eisenbach[3], Samo Penič[4], Lixia Zhang[5], Harshad D Vishwasrao[5], Bahareh Behkam[2], Konstantinos Konstantopoulos[6], Hari Shroff[5,7], Aleš Iglič[4], Elior Peles[3], Amrinder S. Nain[2c], and Nir S Gov[1d]

[1]*Department of Chemical and Biological Physics, Weizmann Institute of Science, Rehovot 7610001, Israel*
[2]*Department of Mechanical Engineering, Virginia Tech, Blacksburg, VA 24061, US*
[3]*Department of Molecular Cell Biology, Weizmann Institute of Science, Rehovot 7610001, Israel*
[4]*Laboratory of Physics, Faculty of Electrical Engineering, University of Ljubljana, Ljubljana, Slovenia*
[5]*Advanced Imaging and Microscopy Resource, National Institutes of Health, Bethesda, Maryland, USA*
[6]*Department of Chemical and Biomolecular Engineering, Johns Hopkins University, Baltimore, MD, USA and*
[7]*Laboratory of High Resolution Optical Imaging, National Institute of Biomedical Imaging and Bioengineering, National Institutes of Health, Bethesda, Maryland, USA*

This PDF file includes:

Supplementary Text

Supplementary Secs. 1 to 8

Supplementary Figs. 1 to 10

Supplementary references (1)

1. PRE-MYELINATION AND MYELINATION PROCESS AND KYMOGRAPH

Here we show the 2D images of myelination process and kymograph of the same set up as in the main paper (Supplementary Fig. 1(A-C), Supplementary Movies-5,6). The kymograph shows that the cells leading edge coils and moves forward. This process is simplified in a simple analytic calculation of a spiral shape that is both rotating and extending upwards on a cylindrical fiber (Supplementary Fig. 1(D-E), Supplementary Movie-7). The kymograph of this schematic diagram shows similar behaviour as the original myelination process.

2. RESULTS FOR PASSIVE VESICLE

We present here the results for the adhesion and spreading of protein-free vesicles on cylindrical substrates, for different fiber radius and strength of adhesion (Supplementary Fig. 2). As expected, the adhered area fraction increases strongly with both fiber radius and adhesion energy, with the transition to complete adhesion determined by the balance between the bending and adhesion energies. At large adhered area fractions, the bending energy per vesicle-substrate area balances the adhesion area, with the complete adhesion regime occurring when: $\kappa/R^2 \leq E_{ad}$, shown by the yellow dashed line in Supplementary Fig. 2.

We next show our results with passive curved proteins ($F = 0$) and a given radius of fiber $R = 10\ l_{min}$ in Supplementary Fig. 3. With the introduction of curved passive proteins, the complete adhesion regime is extended to lower adhesion energies, as was observed for flat substrates [1]. For a given small E_{ad}, as we increase protein density ρ, the vesicle gets adhered more and more. For larger E_{ad}, however, the strong adhesion is achieved even without (or small) proteins. The yellow horizontal dashed line is showing the transition given by $\kappa/R^2 \leq E_{ad}$ (for a given R) above which the adhesion energy dominates.

---

[a] raj-kumar.sadhu@curie.fr
[b] Present address: Institut Curie, PSL Research University, CNRS, UMR 168, Paris, France
[c] nain@vt.edu
[d] nir.gov@weizmann.ac.il

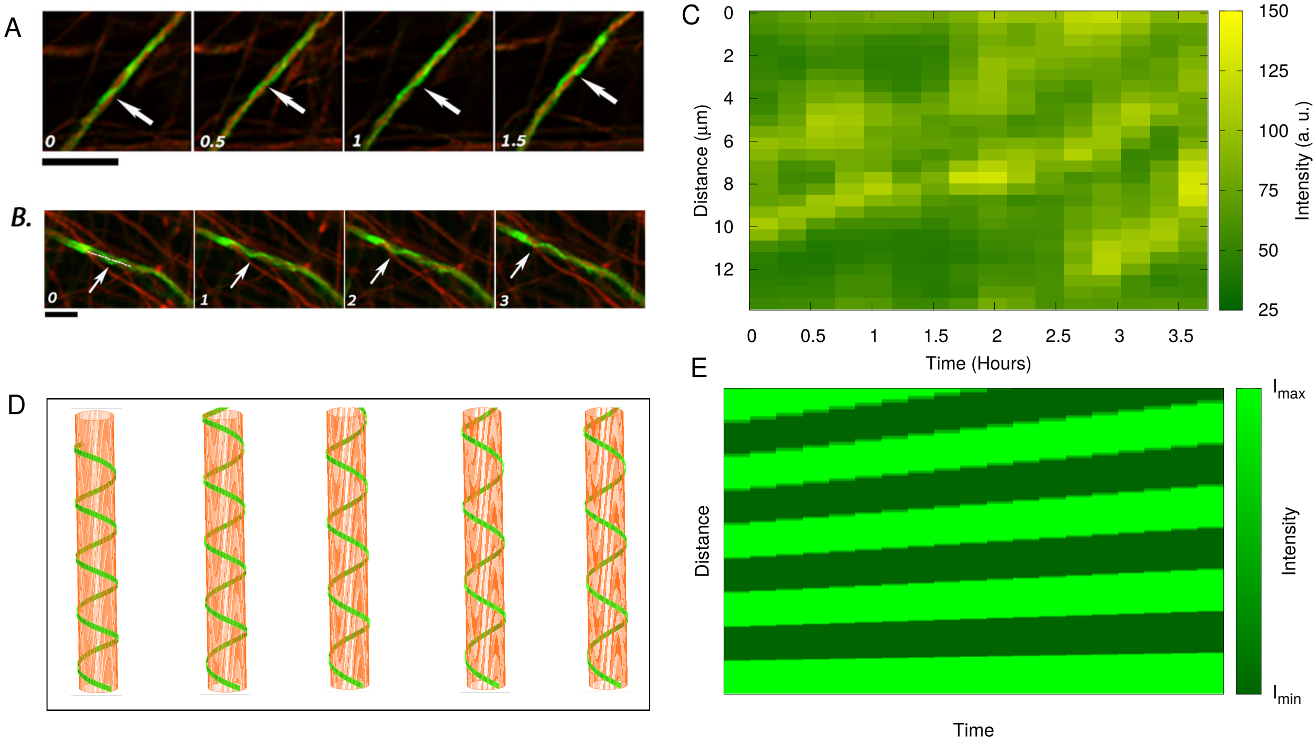

Supplementary FIG. 1. Coiling events involved in inter-cellular interactions during vertebrate myelin development. Time lapse imaging of mouse DRG myelinating culture showing (A) initial contact between a thin process of pre-myelinating Schwann cell (green) spiraling around an axon (red). Time intervals between images are 30 minutes. (B) As myelination proceeds, a thicker Schwann cell process wraps around an axon at a slower speed. Time intervals between images: 1 hour. Arrows point at clear spiral turns, as they rotate. (C) Kymograph showing fluorescence intensity along a fixed line across the spiral shown in (A) (depicted as a dashed white line) over the duration of 4 hours in 15 minute intervals. Scale bars: 10 μm. The kymograph clearly shows the apparent upwards motion of the fluorescent Schwann cell process, as the spiral shape rotates around the axon. (D) A simple analytic calculation of a spiral shape that is both rotating and extending upwards on a cylindrical fiber. (E) Kymograph of the effective intensity of the observed color of the spiral, using spiral calculated in panel D, showing similar behaviour compared to the dynamics of the myelination membrane spiral in panel C.

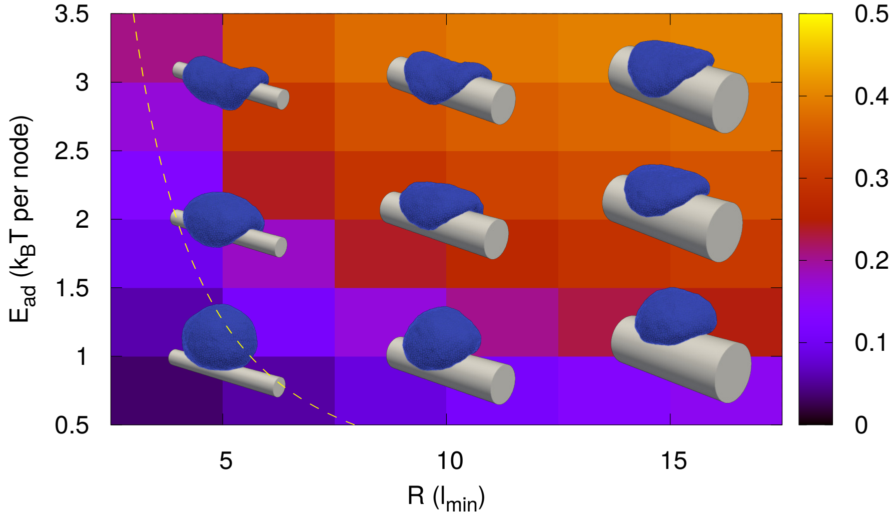

Supplementary FIG. 2. Phase diagram $E_{ad} - R$ plane for protein-free case ($\rho = 0$). Background color is showing the adhered fraction of vesicle. Adhered fraction increases either with R or E_{ad}.

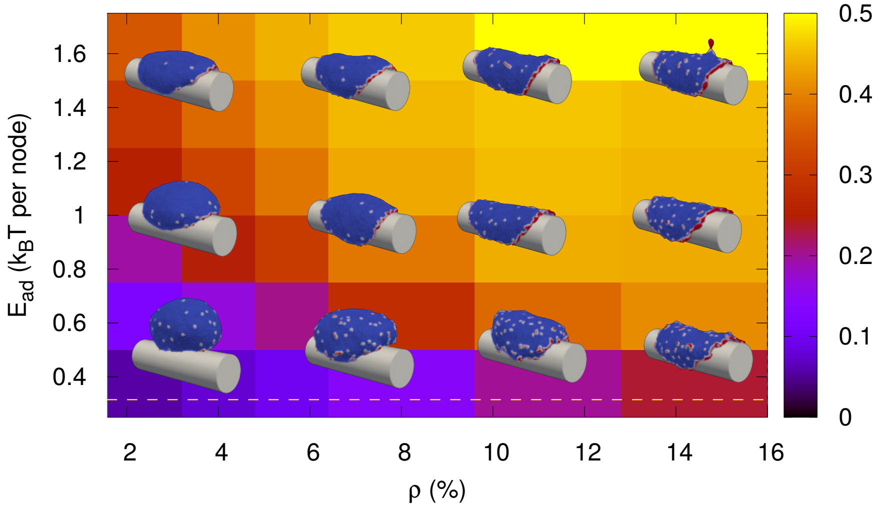

Supplementary FIG. 3. Phase diagram $E_{ad} - \rho$ plane for vesicle with passive proteins ($F = 0$) for a given radius of fiber ($R = 10\ l_{min}$). Background color is showing the adhered area fraction. The adhered fraction increases with E_{ad} and ρ.

3. ANALYTICAL ESTIMATION OF TRANSITION LINES

Here, we present our simple analytical estimation of all the transition lines separating different phases (shown in Fig. 4d, main text).

A. Transition to a pancake shape (phase-I to II or phase-IV to III)

Let us consider the surface area of the vesicle is A. If we make this a flat pancake-like, the radius of this circular face will be given by the relation,

$$\pi R_p^2 \simeq A/2 \tag{1}$$

or,

$$R_p \simeq \sqrt{A/2\pi} \tag{2}$$

The perimeter of the circular face of the pancake-like shape is,

$$2\pi R \simeq \sqrt{2\pi A} \tag{3}$$

If the average size of a protein is, $l_{av} = (l_{min} + l_{max})/2$, then, the number of proteins that will fit in that perimeter is given by,

$$N_p \simeq \frac{\sqrt{2\pi A}}{l_{av}} \tag{4}$$

Now, the average surface area of the vesicle is,

$$A = 2N\frac{\sqrt{3}}{4}l_{av}^2 \tag{5}$$

where, N is the number of nodes, and $2N$ is the approximate number of triangles. Since, $N = 3127$, and $l_{av} = 1.35\ l_{min}$, the total number of proteins that will fit into the rim of circular pancake is,

$$N_p = \frac{\sqrt{2\pi A}}{l_{av}} \simeq 130 \tag{6}$$

The density of proteins is thus,

$$\rho_p(\%) = (100 \times 130)/N \simeq 4\% \tag{7}$$

B. Transition from "hanging" pancake (phase-II) to "wrapped" pancake (phase-III)

Let us assume the vesicle forms a circular pancake-like shape with the radius of R_p, such that the total area of the vesicle, $A = 2\pi R_p^2$. Now, consider the case where the pancake just touching the cylinder and remains unadhered. The adhesion energy in this case is,

$$W_A = -E_{ad}\frac{2dR_p}{l_{av}^2} \tag{8}$$

Here, l_{av} is the average length of the bond, $l_{av} = (l_{min} + l_{max})/2$. Here, we assume that the width that is in contact with the cylinder is of the length $d = l_{min}$, such that the number of adhered nodes are $2R_p/l_{av}^2$. After the pancake is fully adhered, the new adhesion energy and the bending energy are,

$$W_A = -E_{ad}\frac{\pi R_p^2}{l_{av}^2} \tag{9}$$

and the extra bending energy cost for this transition,

$$W_b = \kappa\pi R_p^2 \frac{1}{R^2} \tag{10}$$

The condition for the transition is,

$$\frac{E_{ad}}{l_{av}^2}(\pi R_p - 2) = \kappa\frac{\pi R_p}{R^2} \tag{11}$$

which gives,

$$R^2 = \frac{\kappa}{E_{ad}}\frac{l_{av}^2}{\left(1 - \frac{2}{\pi R_p}\right)} \tag{12}$$

Assuming $R_p \gg 1$, we have,

$$R \sim \sqrt{\kappa/E_{ad}}\, l_{av} \sim 6 l_{min} \tag{13}$$

C. Coiling transition (phase-I to phase-IV)

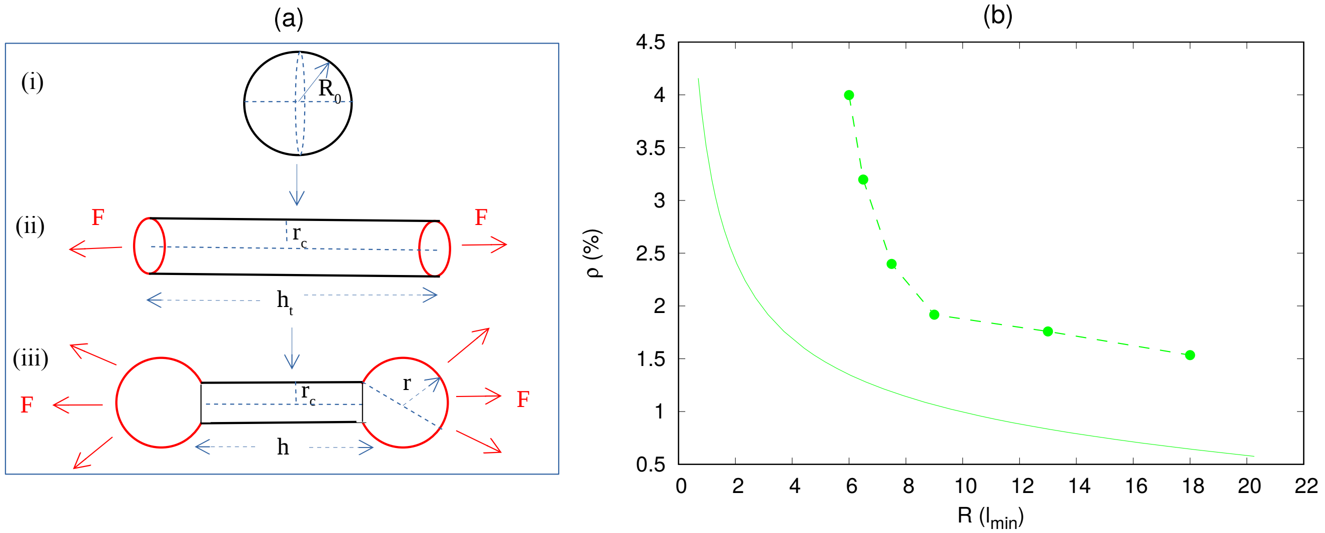

Supplementary FIG. 4. Analytical estimation of transition line for coiling transition (phase-I to phase-IV). (a) Schematic representation of steps of analytical calculations. (i) For the vesicle without any force, the shape is spherical. (ii) For small R, when force is applied, the vesicle shape changes to cylindrical one. (iii) For larger R, the vesicle shape changes to coiling shape, having a combination of cylindrical and circular shape. The red lines are representing the curved proteins. (b) Comparison of analytical estimation with the simulation. Other simulation parameters are same as in Fig. 4, main text.

For small ρ, the vesicle either forms elongated shapes axially aligned, or coiled shape. For small R, the vesicle aligned along the axial direction and for large R, it forms a circumferential (coiled) shape. Let us assume a spherical vesicle having zero force (Supplementary Fig. 4a(i)). As the force increases, the vesicle will get stretched (Supplementary Fig. 4a(ii)). Using the energy minimization, and area conservation, we can get the elongated shape, assuming it to be cylindrical. The area conservation states,

$$4\pi R_0^2 \simeq 2\pi r_c h_t \tag{14}$$

where, we neglect the area for the two circular edges. Now, assume that for spherical vesicle, only a single vertex of adhered, while for cylindrical shape, a linear chain of vertices are adhered along the axial direction. Thus, from energy minimization,

$$(N_c R_0 F + E_{ad} - 8\pi\kappa) r_C^2 - 2R_0^2(E_{ad}/l_{av} + N_c F/2) - 8\pi\kappa R_0^2 (N_c R_0 F + E_{ad} - 8\pi\kappa) = 0 \tag{15}$$

The solution of this equation will give two values of r_c among which, one is greater than R_0 and hence is unphysical. We consider the other solution We can also calculate h_t from area conservation.

Now, let us assume that for large R, when the vesicle goes to a coiled shape, the value of r_c remains same, and the shape becomes a combination of cylinder and two flat circular shapes (Supplementary Fig. 4a(iii)). The radius of the flat circular shapes will be determined by the protein density,

$$2\pi r - 2r_c = N_c l_{av}/2 \tag{16}$$

or,

$$r = (N_c l_{av}/2 + 2r_c)/2\pi \tag{17}$$

From area conservation,

$$4\pi R_0^2 = 2\pi h r_c + 4\pi r^2 \tag{18}$$

and from energy minimization,

$$-E_{ad}h_t/l_{av} + \frac{\kappa}{2}2\pi r_c h_t \frac{1}{r_c^2} = -E_{ad}(h/l_{av} + 2\pi r^2/l_{av}^2) + \frac{\kappa}{2}2\pi r_c h \frac{1}{r_c^2} + \frac{\kappa}{2}4\pi R_0^2 \frac{1}{R^2} \tag{19}$$

$$R^2 = \frac{\kappa A}{2}[E_{ad}(h/l_{av} + 2\pi r^2/l_{av}^2 - h_t/l_{av}) + \pi\kappa/r_c(h_t - h)]^{-1} \tag{20}$$

where, R is the radius of the cylinder. We solve the above equation numerically in the $R-\rho$ plane, and show in Supplementary Fig. 4(b) with green solid line, and compare with simulation results with dashed line-points.

4. QUANTIFICATION OF AXIAL AND CIRCUMFERENTIAL ALIGNMENT

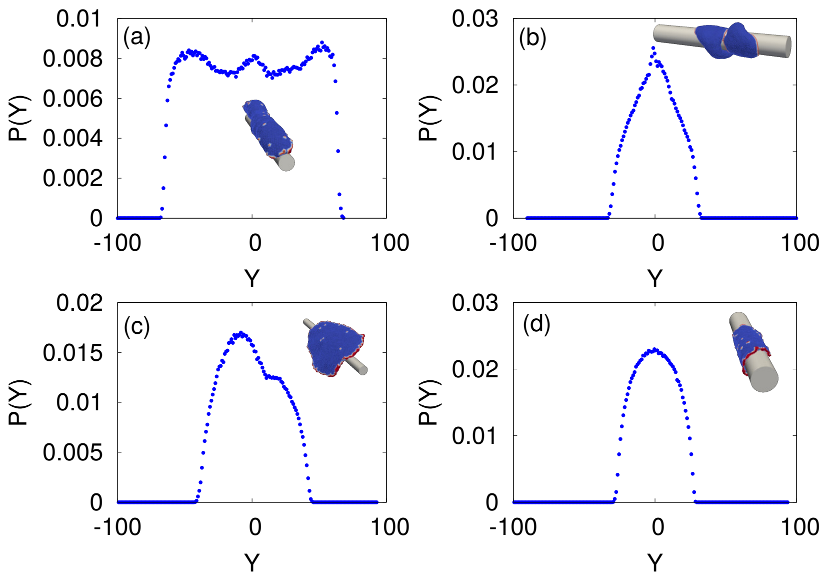

Supplementary FIG. 5. Distribution of vertices along the axial direction. (a) $\rho = 3.2\%$ and $R = 3.0l_{min}$. (b) $\rho = 3.2\%$ and $R = 8.0l_{min}$. (c) $\rho = 9.6\%$ and $R = 3.0l_{min}$. (d) $\rho = 9.6\%$ and $R = 8.0l_{min}$.

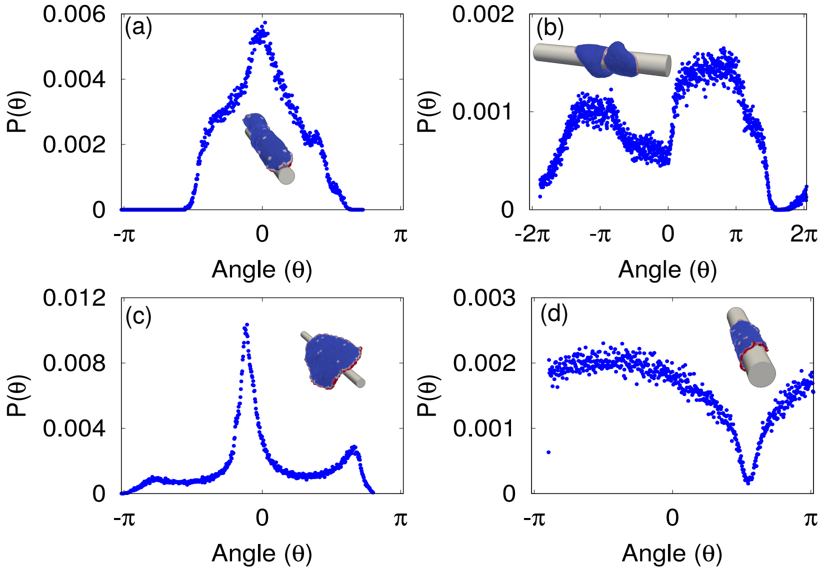

Supplementary FIG. 6. Angular distribution of the vertices along the circumferential direction. (a) $\rho = 3.2\%$ and $R = 3.0l_{min}$. (b) $\rho = 3.2\%$ and $R = 8.0l_{min}$. (c) $\rho = 9.6\%$ and $R = 3.0l_{min}$. (d) $\rho = 9.6\%$ and $R = 8.0l_{min}$.

In our main text, we quantify the axial and circumferential alignment of the vesicle by measuring the variance in the distribution of vertices along the axial direction and the distribution of angle along the circumferential direction respectively. Here, we show the full distribution of the vertices along the axial and circumferential direction in Supplementary Fig. 5 and Supplementary Fig. 6 respectively.

5. CONTRIBUTION OF PROTEIN-PROTEIN BINDING ENERGY IN PROCESS OF AXIAL TO COILING TRANSITION

Here, we show the contribution of protein-protein binding energy in the coiling process in Supplementary Fig. 7. Since the cluster sizes remain almost constant, this contribution is negligible in comparison to other energy contributions.

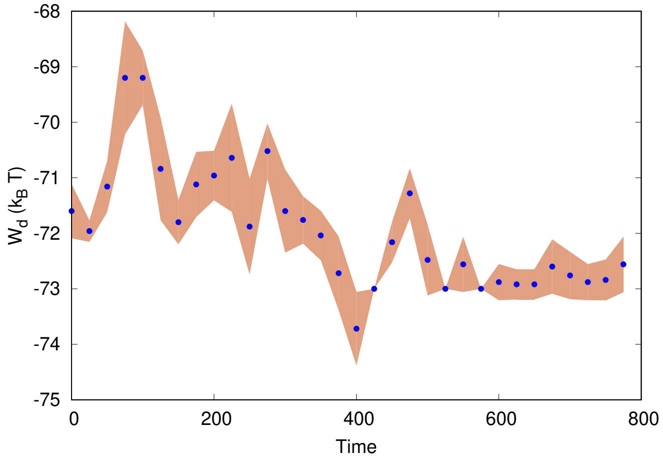

Supplementary FIG. 7. Contribution of protein-protein binding energy in the coiling process. The unit of time is 2×10^4 MC steps. Other parameters are same as in Fig. 5(b-e) of the main text. Data are presented as mean values ± SD.

6. REORIENTATION PROCESS: ENERGY WITH TIME WITHOUT SCALING PER VERTEX

Here, we show the adhesion and bending energies for an arc without scaling by the total number of vertices that are forming the arc, in Supplementary Fig. 8(a-b). We also show the energy of the rest of the cylindrical part of the vesicle (after subtracting the contribution due to the other two arcs) in Supplementary Fig. 8(c-d). We note that the adhesion energy is increasing throughout and bending energy is also larger in the final configuration.

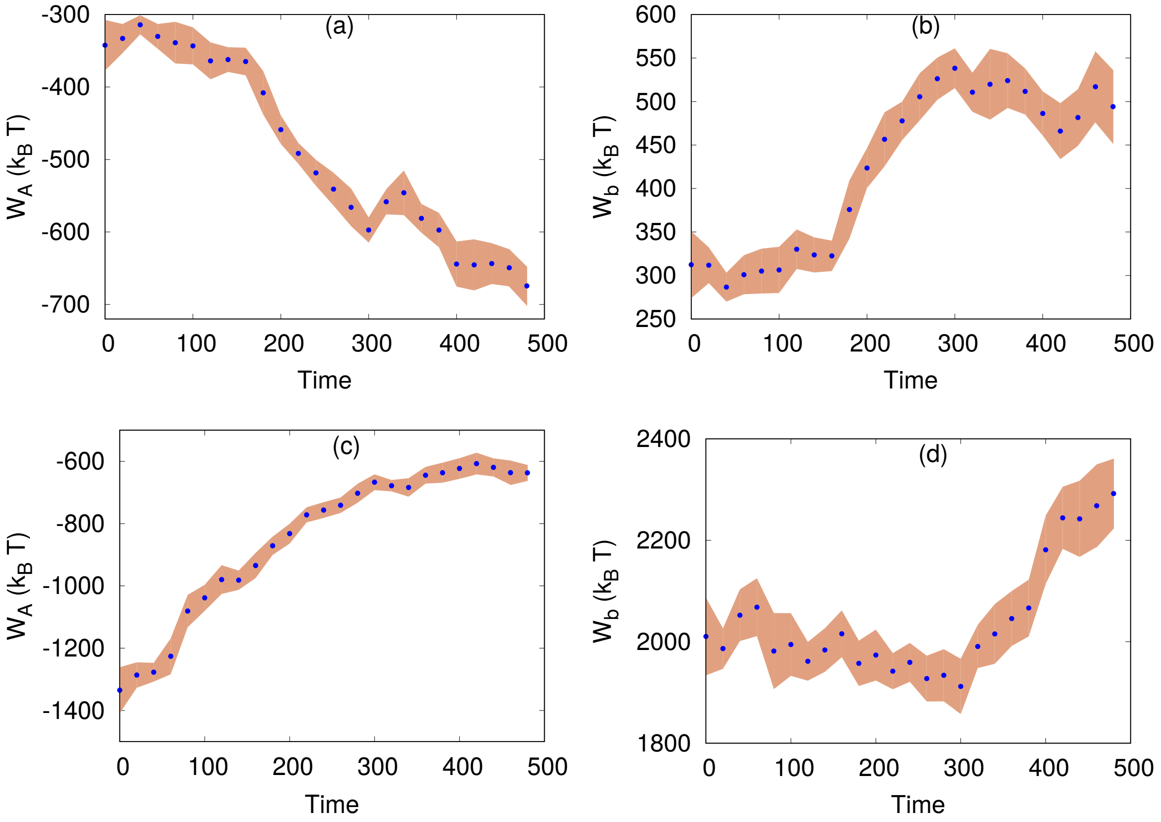

Supplementary FIG. 8. (a) Adhesion energy with time for an arc. (b) Bending energy with time for an arc. (c) Adhesion energy with time for the rest cylindrical part. (d) Bending energy with time for the rest cylindrical part. Other simulation parameters are same as in Fig. 5, main text. Data are presented as mean values ± SD.

7. COILING SPEED FOR DIFFERENT FIBER RADIUS

In Supplementary Fig. 9 we compare the coiling velocity of the leading edge protrusion measured from experiments with the simulation data. The simulation data indicates that over the fiber radii that we can test, the coiling speed does not change significantly (slopes of the graphs in Supplementary Fig. 9b). However, in the experiments the coiling speed was found to decrease for increasing radii, most significantly for small radius (Supplementary Fig. 9d).

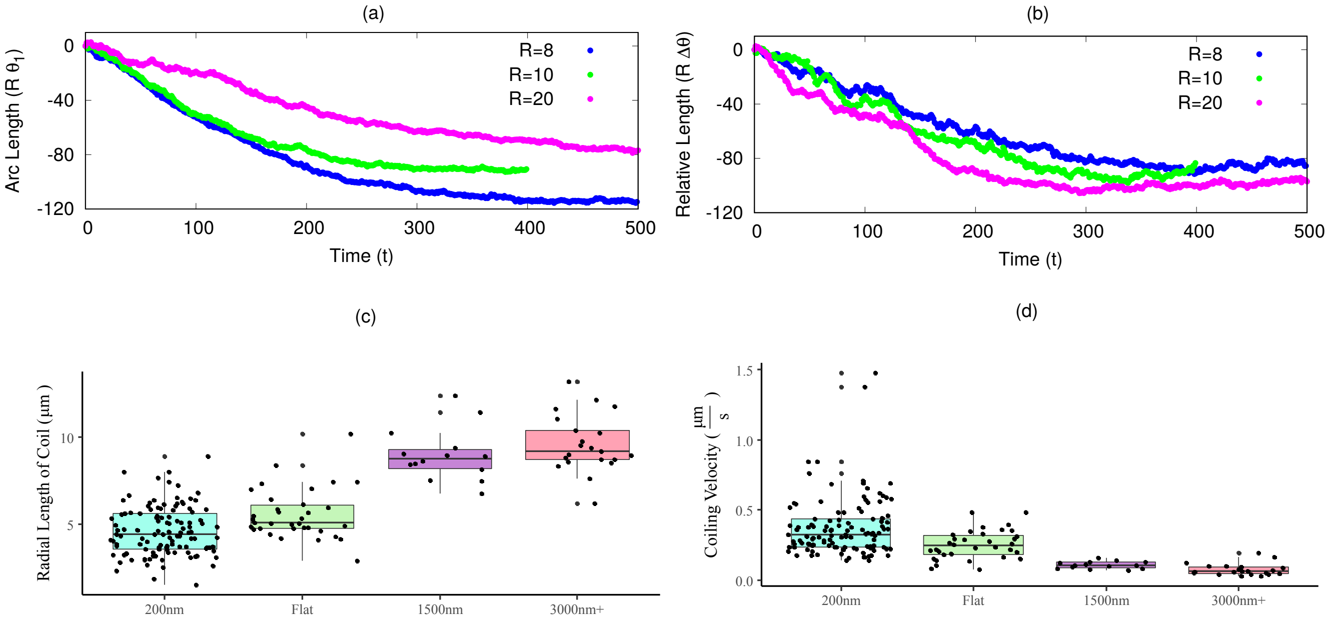

Supplementary FIG. 9. Coiling speed for different fiber radius in simulations, and compared with experiments. (a) The overall angular-length-span of the vesicle coil as function of time. (b) The displacements of the leading-edge arcs along the angular direction, as function of time. (c) Radial length of coil trajectory measured from experiments. Statistics for the boxplot are, 200nm: 2.70 minima, 5.13 median, 8.90 maxima, the number of independent experiments n=65; Flat Ribbon: 2.95 minima, 5.33 median, 10.16 maxima, n=29; 1500nm: 6.79 minima, 8.91 median, 12.38 maxima, n=10; 3000nm: 6.19 minima, 8.98 median, 13.16 maxima, n=16. (d) Coiling velocity from the experimental data, for each of the four cases. Statistics for the boxplot are, 200nm: 0.05 minima, 0.06 median, 0.25 maxima, n=65; Flat Ribbon: 0.04 minima, 0.13 median, 0.2 maxima, n=29; 1500nm: 0.04 minima, 0.06 median, 0.09 maxima, n=10; 3000nm: 0.02 minima, 0.06 median, 0.28 maxima, n=16.

8. COILING WITH VARIOUS STRENGTH OF ADHESION

We study the effect of varying adhesion strength on the coiling of cellular protrusion in our simulation (Supplementary Fig.10(A-B)). When we start with reduced adhesion strength from the beginning ($E_{ad} = 0.50\ k_BT$, Supplementary Fig.10A), the vesicle does not coil around the fiber, but rather remains elongated along the fiber axis, similar to the case of small fiber radius (regime I in Fig.4, main text). Even when we start with a state where the vesicle is already coiled around the fiber (due to high initial adhesion strength) and then suddenly reduce the adhesion strength (Supplementary Fig.10B), the vesicle uncoils.

To test these theoretical predictions we performed experiments using diSPIM for different concentrations of fibronectin coating of the fibers, with solutions of 1, 4, and 16 $\mu g/mL$. Fibers were incubated at $37°C$ with respective solutions for 1 hour in petri dishes. Subsequently, trypsinized cells in media suspension were seeded onto the scaffolds and allowed to spread for 30 mins. Next, media was aspirated and replaced with $50mL$ of live imaging media to begin volume scans in the diSPIM. $488nm$ laser power was between $100 - 300\mu W$ and $561nm$ laser power was between $50 - 150\mu W$.

In the experiments we observed coiling even for the smaller fibronectin density (1 $\mu g/mL$) (Supplementary Fig.10(C-E), Supplementary Movie-24) or even in the absence of any coating of the plastic fibers. It turns out that cells are able to secrete some minimal level of coating which enables them to spread and coil even on bare plastic fibers. We do find however that the persistence of coiling events was reduced when the adhesion strength was decreased (Supplementary Fig.10C). On the fiber of large radius the ruffles move more randomly compared to the 4 $\mu g/mL$ system (lower value of the MSAD exponent α in Supplementary Fig.10D,E), which may also indicate some reduced coiling tendency.

At high levels of adhesion, the ruffles become more strongly adsorbed to the fiber, making it very difficult to accurately follow their coiling motion in the experiment (Supplementary Fig.10C-E).

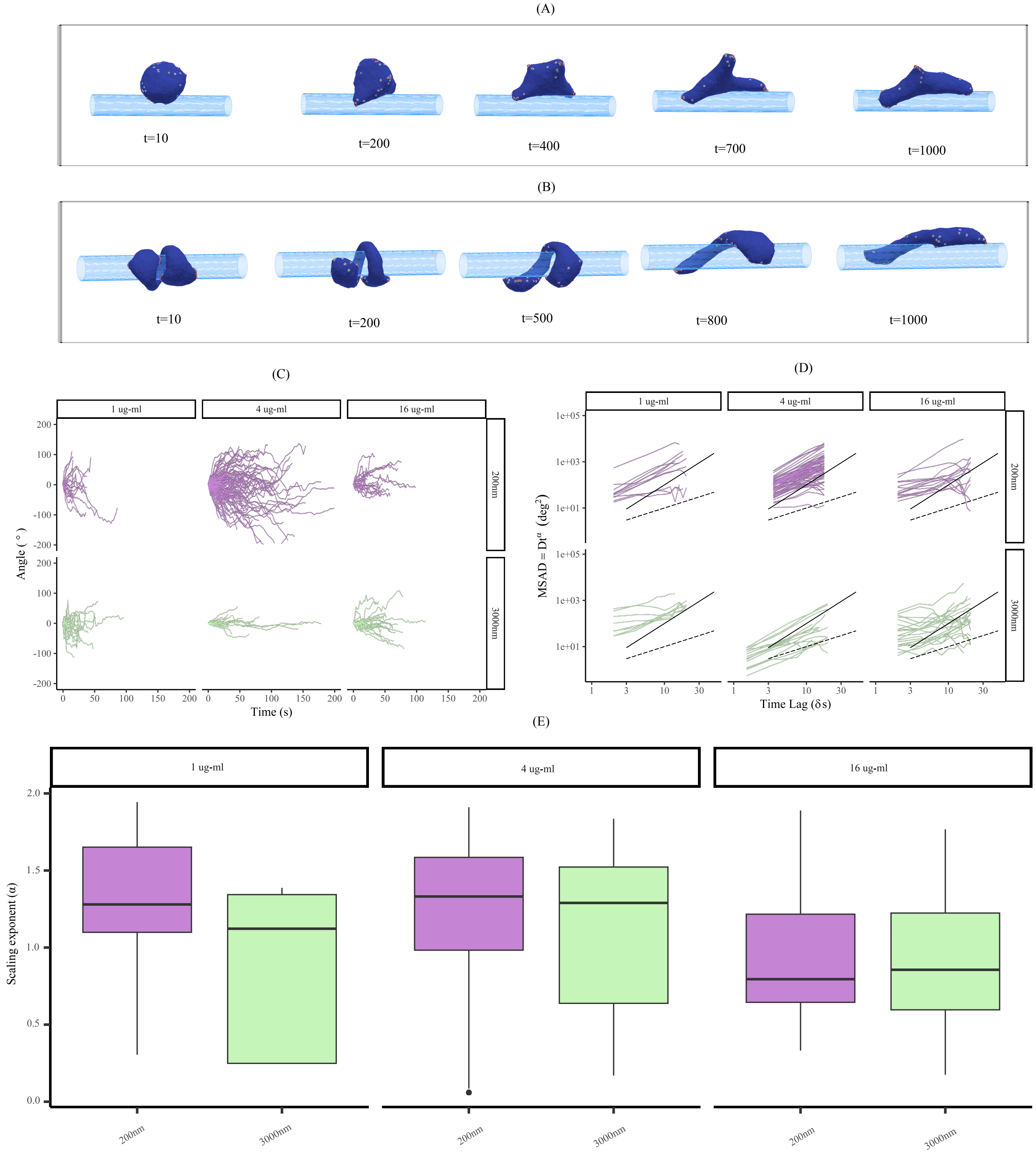

Supplementary FIG. 10. Coiling with various strength of adhesion (in simulation) and fibronectin coating density (in experiment). (A) We start with a spherical vesicle with reduced adhesion, and follow the evolution in time. (B) A vesicle in the coiling state is used as an initial condition, and the adhesion strength is then reduced, which leads to de-coiling. (C) Angular position of the leading edge protrusions with time for different density of fibronectin and two values of the fiber radius. The ruffles in the low fibronectin coating concentration are less persistent, while for the high coating concentration the ruffles are strongly adhered to the fiber and are difficult to observe. (D) Mean square angular displacement (MSAD) for the same cases as shown in (C). (E) The value of the power-law exponent (α) from the experimental MSAD (D). The ruffles on the large radius and low fibronectin coating concentration are moving more randomly, while their trajectories on the large fibronectin coating concentration are difficult to observe with good accuracy. For simulations, we use reduced adhesion strength of $E_{ad} = 0.50\ k_BT$, and other parameters are same as in Fig. 5, main text. Statistics for the boxplot are, $1\mu g/ml$ 200nm: 0.30 minima, 1.25 median, 1.94 maxima, n=11; $1\mu g/ml$ 3000nm: -0.52 minima, 0.84 median, 1.39 maxima, N = 9; 4 $\mu g/ml$ 200nm: -0.89 minima, 1.19 median, 1.91 maxima, N = 65; 4 $\mu g/ml$ 3000nm: -0.04 minima, 0.43 median, 0.89 maxima, n=16; 16 $\mu g/ml$ 200nm: -0.53 minima, 0.79 median, 1.89 maxima, n=15; 16 $\mu g/ml$ 3000nm: -0.94 minima, 0.80 median, 1.77 max, n=22.

Supplementary references

[1] Raj Kumar Sadhu, Samo Penič, Aleš Iglič, and Nir S. Gov. Modelling cellular spreading and emergence of motility in the presence of curved membrane proteins and active cytoskeleton forces. *The European Physical Journal Plus*, 136(5):495, May 2021.
