# Supplementary material for: Experimental and theoretical model for the origin of coiling of cellular protrusions around fibers
Source: Nat Commun. 2023 Sep 12;14:5612. doi: 10.1038/s41467-023-41273-y (PMC10497540; doi:10.1038/s41467-023-41273-y)
Supplement: Supplementary file 3 — Description of Additional Supplementary Files [file 41467_2023_41273_MOESM3_ESM.pdf]

Description Of Additional Supplementary Files

Supplementary Movie-S1: Experimental movie corresponding to Fig. 1. Deconvolved isometric render in ImageJ 3D Viewer of a C2C12 expressing GFP actin spread on a rhodamine fibronectin coated 200nm fiber. Scale bar shown is for 10 µm.

Supplementary Movie-S2: Experimental movie corresponding to Fig. 1. Zoomedin perspective of leading/trailing edge of the cell as it migrates and coils around the 200nm fiber. Scale bar shown is for 5 µm.

Supplementary Movie-S3: Experimental movie corresponding to Fig. 1. Zoomedin perspective of leading/trailing edge of the cell as it migrates and coils around the 200nm fiber. Scale bar shown is for 5 µm.

Supplementary Movie-S4: Rotation series of 3D rendered images based on a particular frame of time lapse imaging of mouse DRG myelinating culture showing initial contact between a premyelinating Schwann cell (green) and an axon (red).

Supplementary Movie-S5: Time lapse imaging of mouse DRG myelinating culture showing initial contact (arrow) between a pre-myelinating Schwann cell (green) and an axon (red). Images were taken at time intervals of 15min. The Schwann cell process spirals around the axon at a speed of aprx 90 minutes per round .

Supplementary Movie-S6: Time lapse imaging of mouse DRG myelinating culture, taken time intervals of 15 minutes, showing a myelinating Schwann cell membrane (green, arrow) wrapping around an axon (red) at a speed of aprx 180 minutes per round, generating a prominent dynamic spiral.

Supplementary Movie-S7: Movie showing the dynamics of the leading edge protrusions on a fiber which is both rotating and extending upwards using simple analytic calculation.

Supplementary Movie-S8: Spreading of proteinfree vesicle on cylindrical fiber of smaller radius. The parameter values are : Ead=1.5 kBT, R=5.0 lmin.

Supplementary Movie-S9: Spreading of proteinfree vesicle on cylindrical fiber of larger radius. The parameter values are : Ead=1.5 kBT, R=15.0 lmin.

Supplementary Movie-S10: Spreading of a vesicle with passive proteins (low density) on cylindrical fiber. The parameters are: Ead= 1.0 kBT, ρ=1.6%, R= 10.0 lmin.

Supplementary Movie-S11: Spreading of a vesicle with passive proteins (medium density) on cylindrical substrate. The parameters are: Ead= 1.0 kBT, ρ=3.2%, R=10.0 lmin.

Supplementary Movie-S12: Spreading of a vesicle with passive proteins (high density) on cylindrical substrate. The parameters are: Ead=1.0 kBT, ρ=6.4%, R=10.0 lmin.

Supplementary Movie-S13: Spreading of a vesicle with active proteins with small R and small ρ (Phase-I). The parameters are: Ead=1.0 kBT, ρ=3.2%, R=4.0 lmin, F=2.0 kBT/ lmin.

Supplementary Movie-S14: Spreading of a vesicle with active proteins with small R and large ρ (Phase-II). The parameters are: Ead=1.0 kBT, ρ=6.4%, R=4.0 lmin, F=2.0 kBT/ lmin.

Supplementary Movie-S15: Spreading of a vesicle with active proteins with large R and large ρ (Phase-III). The parameters are: Ead=1.0 kBT, ρ=6.4, R=10.0 lmin, F=2.0 kBT/ lmin.

Supplementary Movie-S16: Spreading of a vesicle with active proteins with large R and small ρ (Phase-IV). The parameters are: Ead=2.0 kBT, ρ=2.4%, R=10.0 lmin, F=2.0 kBT/ lmin

Supplementary Movie-S17: Axial to circumferential (coiling) transition. The parameters are: Ead=1.0 kBT, ρ=2.4%, R=10.0 lmin, F=2.0 kBT/ lmin

Supplementary Movie-S18: Spreading of vesicle with active proteins on a cylinder with elliptical cross-section. The parameters are: Ead=1.0 kBT, ρ=3.2%, Rx=12.0 lmin, Ry=7.77 lmin, F=2.0 kBT/ lmin

Supplementary Movie-S19: Experimental movie corresponding to Fig. 7, 200nm, leading-edge 1. Maximum intensity projection of the leading-edge volume used for rotational analysis. Dots and lines are overlayed paths using ImageJ manual tracking plugin.

Supplementary Movie-S20: Experimental movie corresponding to Fig. 7, 200nm, leading-edge 2. Maximum intensity projection of the leading-edge volume used for rotational analysis. Dots and lines are overlayed paths using ImageJ manual tracking plugin.

Supplementary Movie-S21: Experimental movie corresponding to Fig. 7, flat ribbon. Maximum intensity projection of the leading-edge volume used for rotational analysis. Dots and lines are overlayed paths using ImageJ manual tracking plugin.

Supplementary Movie-S22: Experimental movie corresponding to Fig. 7, 1500nm. Maximum intensity projection of the leading-edge volume used for rotational analysis. Dots and lines are overlayed paths using ImageJ manual tracking plugin.

Supplementary Movie-S23: Experimental movie corresponding to Fig. 7, 3000 nm. Maximum intensity projection of the leading-edge volume used for rotational analysis. Dots and lines are overlayed paths using ImageJ manual tracking plugin.

Supplementary Movie-S24: Experimental movie corresponding to Fig. S-10 with reduced fibronectin density (1 μg/mL), and fiber radius 200nm.
