# Supplementary material for: Experimental and theoretical model for the origin of coiling of cellular protrusions around fibers
Source: Nat Commun. 2023 Sep 12;14:5612. doi: 10.1038/s41467-023-41273-y (PMC10497540; doi:10.1038/s41467-023-41273-y)
Supplement: Supplementary file 28 — Reporting Summary [file 41467_2023_41273_MOESM28_ESM.pdf]

nature portfolio

Corresponding author(s): Raj Kumar Sadhu
Amrinder S Nain
Nir S Gov

Last updated by author(s): Aug 10, 2023

Reporting Summary

Nature Portfolio wishes to improve the reproducibility of the work that we publish. This form provides structure for consistency and transparency in reporting. For further information on Nature Portfolio policies, see our Editorial Policies and the Editorial Policy Checklist.

Statistics

For all statistical analyses, confirm that the following items are present in the figure legend, table legend, main text, or Methods section.

n/a	Confirmed	
☐	☒	The exact sample size (*n*) for each experimental group/condition, given as a discrete number and unit of measurement
☐	☒	A statement on whether measurements were taken from distinct samples or whether the same sample was measured repeatedly
☐	☒	The statistical test(s) used AND whether they are one- or two-sided<br>*Only common tests should be described solely by name; describe more complex techniques in the Methods section.*
☒	☐	A description of all covariates tested
☐	☒	A description of any assumptions or corrections, such as tests of normality and adjustment for multiple comparisons
☐	☒	A full description of the statistical parameters including central tendency (e.g. means) or other basic estimates (e.g. regression coefficient) AND variation (e.g. standard deviation) or associated estimates of uncertainty (e.g. confidence intervals)
☐	☒	For null hypothesis testing, the test statistic (e.g. *F*, *t*, *r*) with confidence intervals, effect sizes, degrees of freedom and *P* value noted<br>*Give P values as exact values whenever suitable.*
☒	☐	For Bayesian analysis, information on the choice of priors and Markov chain Monte Carlo settings
☒	☐	For hierarchical and complex designs, identification of the appropriate level for tests and full reporting of outcomes
☒	☐	Estimates of effect sizes (e.g. Cohen's *d*, Pearson's *r*), indicating how they were calculated

Our web collection on statistics for biologists contains articles on many of the points above.

Software and code

Policy information about availability of computer code

Data collection	FIJI (ImageJ 1.54f)+Plugin TransformJ (4.1.0), and Micromanager, ASI diSPIM
Data analysis	R (4.1.1), R Studio (2023.06.0+421), Excel, Matlab

For manuscripts utilizing custom algorithms or software that are central to the research but not yet described in published literature, software must be made available to editors and reviewers. We strongly encourage code deposition in a community repository (e.g. GitHub). See the Nature Portfolio guidelines for submitting code & software for further information.

Data

Policy information about availability of data

All manuscripts must include a data availability statement. This statement should provide the following information, where applicable:

- Accession codes, unique identifiers, or web links for publicly available datasets
- A description of any restrictions on data availability
- For clinical datasets or third party data, please ensure that the statement adheres to our policy

All data, spreadsheets, R codes for plotting are available

Human research participants

Policy information about studies involving human research participants and Sex and Gender in Research.

Reporting on sex and gender	N/A
Population characteristics	N/A
Recruitment	N/A
Ethics oversight	N/A

Note that full information on the approval of the study protocol must also be provided in the manuscript.

Field-specific reporting

Please select the one below that is the best fit for your research. If you are not sure, read the appropriate sections before making your selection.

☒ Life sciences ☐ Behavioural & social sciences ☐ Ecological, evolutionary & environmental sciences

For a reference copy of the document with all sections, see nature.com/documents/nr-reporting-summary-flat.pdf

Life sciences study design

All studies must disclose on these points even when the disclosure is negative.

Sample size	Sample size was determined based on previous studies from our group
Data exclusions	We have not explicitly excluded any data from our analysis. However, we set predetermined criteria for analysis which have been reported either in the method section or the result section of the manuscript
Replication	Data collection was done over multiples round of experiments. All replication led to successful experiments. In few isolated cases where the fiber networks were destroyed, the entire experiment was discarded. Data was collected from at least three independent replicates.
Randomization	Section of videos to be analyzed were chosen randomly from sets of experiments
Blinding	Blinding is not relevant for the representation of physically measured events such as coiling ruffles

Reporting for specific materials, systems and methods

We require information from authors about some types of materials, experimental systems and methods used in many studies. Here, indicate whether each material, system or method listed is relevant to your study. If you are not sure if a list item applies to your research, read the appropriate section before selecting a response.

Materials & experimental systems

n/a	Involved in the study	
☒	☐	Antibodies
☐	☒	Eukaryotic cell lines
☒	☐	Palaeontology and archaeology
☐	☒	Animals and other organisms
☒	☐	Clinical data
☒	☐	Dual use research of concern

Methods

n/a	Involved in the study	
☒	☐	ChIP-seq
☒	☐	Flow cytometry
☒	☐	MRI-based neuroimaging

Eukaryotic cell lines

Policy information about cell lines and Sex and Gender in Research

Cell line source(s)	Mouse Muscle Myoblasts (C2C12s) ATCC
Authentication	Cell line was authenticated by ATCC Inc. as specified in the certificate of analysis provided on their website.

Mycoplasma contamination	Cells were mycoplasma free as tested by ATCC and were used as received. ATCC tests for mycoplasma using PCR-based method. ATCC also performs morphology and COI assays for interspecies determination.
Commonly misidentified lines (See ICLAC register)	No commonly misidentified cell lines were used in this study.

Animals and other research organisms

Policy information about studies involving animals; ARRIVE guidelines recommended for reporting animal research, and Sex and Gender in Research

Laboratory animals	C57/BL6 mice expressing S-MAG-GFP under MAG promoter
Wild animals	None
Reporting on sex	Pregnant female at E13.5 days of gestation
Field-collected samples	None
Ethics oversight	Weizmann Institute's Animal Care and Use Committee.

Note that full information on the approval of the study protocol must also be provided in the manuscript.
